# Controlling Nitrogen Doping in Graphene with Atomic Precision: Synthesis and Characterization

**DOI:** 10.3390/nano9030425

**Published:** 2019-03-12

**Authors:** Tomotaroh Granzier-Nakajima, Kazunori Fujisawa, Vivek Anil, Mauricio Terrones, Yin-Ting Yeh

**Affiliations:** 1 Department of Physics, Pennsylvania State University, University park, PA 16802, USA; txg210@psu.edu (T.G.-N.); vja5066@psu.edu (V.A.); mut11@psu.edu (M.T.); 2 Center for 2-Dimensional and Layered Materials (2DLM), Pennsylvania State University, University park, PA 16802, USA; 3 Department of Engineering Science and Mechanics, Pennsylvania State University, University park, PA 16802, USA; 4 Department of Chemistry, and Materials Science and Engineering, Pennsylvania State University, University park, PA 16802, USA; 5 Materials Research Institute, Pennsylvania State University, University park, PA 16802, USA; 6 The Huck Institutes of the Life Sciences, Pennsylvania State University, University park, PA 16802, USA

**Keywords:** nitrogen doping, graphene, bonding configuration, sublattice segregation, dopant segregation

## Abstract

Graphene provides a unique platform for the detailed study of its dopants at the atomic level. Previously, doped materials including Si, and 0D-1D carbon nanomaterials presented difficulties in the characterization of their dopants due to gradients in their dopant concentration and agglomeration of the material itself. Graphene’s two-dimensional nature allows for the detailed characterization of these dopants via spectroscopic and atomic resolution imaging techniques. Nitrogen doping of graphene has been well studied, providing insights into the dopant bonding structure, dopant-dopant interaction, and spatial segregation within a single crystal. Different configurations of nitrogen within the carbon lattice have different electronic and chemical properties, and by controlling these dopants it is possible to either n- or p-type dope graphene, grant half-metallicity, and alter nitrogen doped graphene’s (NG) catalytic and sensing properties. Thus, an understanding and the ability to control different types of nitrogen doping configurations allows for the fine tuning of NG’s properties. Here we review the synthesis, characterization, and properties of nitrogen dopants in NG beyond atomic dopant concentration.

## 1. Introduction

Doping has been used in silicon (Si)-based semiconductor technologies to alter the electronic properties (i.e., carrier density) of Si wafers by substitutionally incorporating non-isoelectronic heteroatoms. Two methods, ion implantation and thermal diffusion, have been used to introduce dopants such as boron, aluminum, arsenic, and phosphorus [1]. For Si wafers, doped heteroatoms distribute non-uniformly, thus forming a dopant concentration gradient with depth. Because of this, a depth profile of the dopant concentration has been used to understand the electronic properties of doped Si wafers.

Similar doping strategies have also been applied to the various forms of carbon nanomaterials such as fullerenes (0D), nanotubes (1D), and graphene (2D), in order to modify their electronic and chemical properties [2,3]. For carbon nanomaterials, dopants have been incorporated during growth (in situ growth doping), or by post-treatment (post growth doping). Because carbon nanomaterials do not have a bulk structure or 3D structural ordering, a uniform incorporation of dopants is expected. Therefore, instead of a dopant concentration gradient varying with depth, a single value for the dopant concentration has been used to understand the effects of doping in carbon nanomaterials [3,4,5,6]. However, the small size and low dimensional structure of 0D and 1D carbon nanomaterials leads to aggregation and atomically resolved details of single dopants and their mutual interactions have not been addressed. In contrast, micro- to millimeter large single crystals are available for the 2D carbon nanomaterial graphene, leading to a unique platform to investigate the fundamentals of single dopants.

**Figure 1:**
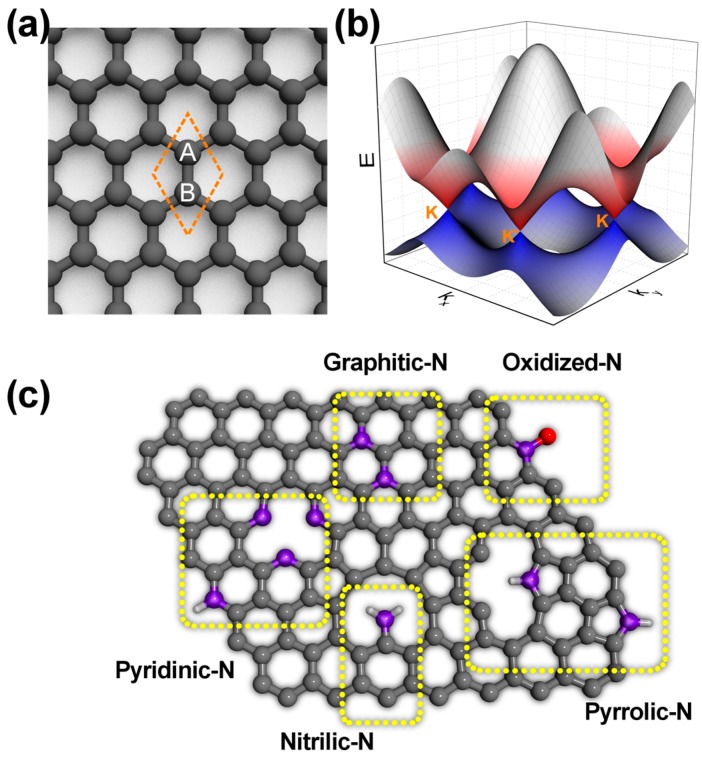
Basics of graphene and nitrogen doped graphene. (**a**) Atomic structure and (**b**) electronic band structure of pristine graphene. The orange diamond in (**a**) illustrates the unit cell which contains two carbon atoms on sublattices A and B. (**c**) Common nitrogen dopant configurations in graphene.

Graphene possesses a two-dimensional structure with sp^2^ hybridized carbon atoms arranged in two intersecting triangle lattices (A and B), in order to form a honeycomb pattern (Figure 1a). Since the conduction band and the valence band exhibit a linear dispersion, and these two bands touch at the K and K′ points forming a Dirac cone (Figure 1b), graphene has shown ultrahigh mobility (~200,000 cm^2^ V^−1^ s^−1^) [2] and broadband optical absorbance (2.3%) in the visible range [5]. Besides these exciting properties, a large Young’s modulus (1TPa) [6], high room temperature thermal conductivity (~5000 W/mK) [3], and high surface area (2630 m^2^/g) [4] have also been reported.

To achieve a wide range of property modifications to graphene, several different heteroatoms—boron [7], nitrogen [8], phosphorus [9], sulfur [10], chlorine [11], fluorine [12], bromine [11], silicon [13], and germanium [14]—have been used as dopants. Depending on the chemistry of the selected element, different heteroatoms bond to carbon atoms differently, thus leading to distinct dopant bonding configurations within graphene. Because they have similar atomic radii and chemical bond lengths, neighboring elements boron (B) and nitrogen (N), can easily substitute carbon in the graphene lattice, leading to p- and n-type doping, respectively. However, elements with relatively larger atomic radii (e.g., silicon and germanium), replace one or two carbon atoms and more electronegative halogens form sp^3^-like bonding with graphene. Although most studies on heteroatom doped graphene only consider dopant concentration rather than dopant bonding configuration, different dopant bonding configurations have been reported for N-doped graphene (NG) [3,15]. In NG, nitrogen is not observed only in a simple substitutional configuration (graphitic-N) but can also be accompanied by a vacancy (pyridinic-N), form a five-membered ring (pyrrolic-N), triple bond to a carbon atom at a zigzag edge (nitrilic-N), and partially oxidize (oxidized-N) (Figure 1c). These different dopant configurations affect the local charge distribution and local density of states differently leading to different electronic, catalytic, and sensing properties [2,3]. Owing to the 2D structure of graphene and its monatomic thickness there is no dopant concentration gradient. Therefore, further detailed investigations such as imaging-based quantification of dopant configurations, long-range interactions, and the spatial distribution of nitrogen dopants, are possible in NG. Interestingly, dopants in NG have been observed to occupy a single triangle sublattice of graphene [16,17,18,19] (Figure 1c), as well as spatially segregate within a single crystal domain [20,21].

The ability to control the finer properties of nitrogen dopants beyond the dopant concentration allows their unique properties to be utilized and expands and enables the finer tuning of NG for various electronic [22,23], catalytic [24,25,26,27], and spintronic applications [28,29]. Although several comprehensive reviews have discussed preparation of nitrogen containing carbon nanomaterials and their applications [3,4,5,6], focus has not yet been given to characterizing single dopants. In this review, we highlight recent progress on the understanding and control of nitrogen dopants in NG, to help readers obtain a big-picture view of dopant control in graphene. We provide an overview of its synthesis by categorizing in situ growth and post-growth treatments with aspects of dopant control. Here, high quality monolayer graphene that is produced by mechanical exfoliation and chemical vapor deposition techniques are discussed.

## 2. Preparation of Nitrogen Doped Graphene

Nitrogen is the most well-studied dopant in graphene. Because nitrogen is next to carbon in the periodic table, and the N–C bond length is comparable to the C–C bond, nitrogen doping leads to a relatively small distortion of the graphene lattice making it an ideal candidate for doping. There are three main dopant bonding configurations observed experimentally when nitrogen is doped in graphene (Figure 1c): *graphitic-N*, where nitrogen bonding to three carbon atoms directly substitutes a carbon atom; *pyridinic-N*, where a nitrogen atom is accompanied by a vacancy and bonds to two carbon atoms as part of a six-membered ring; and *pyrrolic-N*, where nitrogen bonds to two carbon atoms as part of a five-membered ring. Each dopant bonding configuration can have different catalytic activities and affect the electronic structure in different ways. Therefore, the ability to engineer NG to have the desired dopant configuration will allow control over device sensitivity and improve reproducibility.

**Figure 2:**
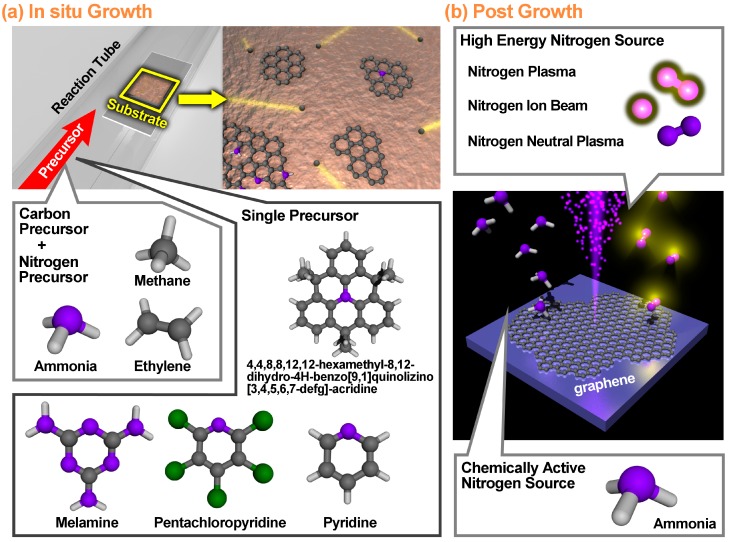
Schematic illustration of preparation methods of nitrogen doped graphene (NG). (**a**) in situ growth doping of nitrogen atoms via chemical vapor deposition (CVD) process. Chemicals which contain either carbon or nitrogen have been used as C- or N-precursor. (**b**) Post growth treatment to incorporate nitrogen into graphene lattice. For, post growth treatment, high energy nitrogen source and chemically active nitrogen source are used.

NG may be prepared either during the growth of graphene itself or afterwards as a post treatment of pristine graphene (Figure 2). When graphene is doped during synthesis, a nitrogen containing precursor is introduced into the system during the growth, and nitrogen gets incorporated into the lattice. In order to dope graphene after its initial growth, a defect site needs to be created and then a nitrogen atom needs to take its place. This is usually done by exposure to a high energy and chemically active nitrogen source such as nitrogen plasma [30,31,32,33] or ammonia (NH_3_) at high temperatures [17,34]. Dopant bonding configuration control can be achieved with both synthesis methods.

### 2.1. In Situ Growth Doping

There are several ways to grow NG in situ. Chemical vapor deposition (CVD) is a widely used technique for the growth of high-quality NG. In general, a CVD growth involves the flow of nitrogen and carbon containing precursors through a furnace onto a target substrate, heated to a desired temperature (Figure 2a). The precursor can either be gas phase as in the case of ammonia (NH_3_) [18,35,36,37,38]; sublimated solid phase, such as melamine [26,39]; or liquid phase, such as pyridine [16]. Several metallic substrates have also been used including Cu, Ni, and Pt. Cu is a common choice for growth substrate because its low carbon solubility prevents growth once there is no more bare Cu [40]. This surface mediated growth promotes monolayer growth and inhibits multilayer growth. Nearly all parameters including the choice of precursor, flow rate, temperature, pressure, and growth substrate can control the final dopant percentage and bonding configuration. Common choices for gas phase precursors include methane (CH_4_) and ammonia (NH_3_) as carbon and nitrogen containing gases, respectively [18,35,36,37,38]. When the nitrogen bonding configuration of ammonia-grown NG samples is investigated with X-ray photoelectron spectroscopy (XPS), most of the nitrogen is found to be in the graphitic-N configuration [18,35,36,38], although there are accounts [37] of the nitrogen being in the pyridinic-N and pyrrolic-N configurations.

Although several methods can achieve control of the bonding configuration, beyond the use of methane and ammonia as precursors which primarily lead to graphitic N, large scale trends which predict the type of dopant bonding configuration before growth and characterization are not easily identifiable. Table 1 compiles different in situ NG synthesis methods and the resulting nitrogen bonding configuration. The effect of the choice of precursor while growing NG was studied by Imamura et al. who separately synthesized NG by using two different nitrogen and carbon containing precursors, pyridine (C_5_H_5_N) and acrylonitrile (C_3_H_3_N) [41]. Although both precursors contain both carbon and nitrogen, only pyridine produced NG while acrylonitrile produced pristine graphene. The underlying growth mechanism that these authors proposed relies on molecule decomposition and diffusion on the growth substrate, in this case Pt(111). Acrylonitrile (C_3_H_3_N) contains C–C single, C=C double, and C≡C triple bonds. When exposed to elevated temperatures the relatively weak C–C single bonds are preferentially broken creating C≡N fragments. These fragments are then removed from the Pt(111) surface by forming volatile C_2_N_2_ or HCN molecules, and pristine graphene is created [41]; when pyridine (C_5_H_5_N) is used as the precursor at relatively low temperatures, this does not happen and NG is produced. A similar analysis was performed by Katoh et al. [42]; the authors grew NG with four aromatic nitrogen-containing precursors, quinoline (C_9_H_7_N), pyridine (C_5_H_5_N), pyrrole (C_4_H_5_N), and pyrimidine (C_4_H_4_N_2_) on Pt(111) at a relatively low temperature of 500 °C. They found that the aromatic molecules that had the highest activation energies for the breaking of the aromatic ring had the highest dopant concentration. The explanation is again based on precursor decomposition and volatile molecule formation. The precursors which have lower activation energies are more likely to break, and upon breaking they form volatile molecules such as HCN which take nitrogen away from the growth substrate, thus leading to lower dopant concentrations. The authors also find that the bonding configuration of the nitrogen dopant in the graphene lattice tended to reflect the bonding configuration in the source molecule. Nitrogen dopants in quinoline and pyrrole derived NG tended to occupy pyridinic and pyrrolic sites respectively. However, nitrogen in pyridine and pyrimidine derived NG tended to adopt pyridinic- and graphitic-N dopant sites, as a result of their already hexagonal molecular shape. These analyses provide a first step towards the understanding and prediction of the nitrogen dopant bonding configuration in NG as a function of source molecules.

### 2.2. Post Growth Doping

The process for doping pristine graphene after its initial growth involves exposing pristine graphene to a relatively high energy and chemically active nitrogen source in order to create defects in the graphene lattice and replace the carbon with nitrogen atoms (Figure 2b). Table 2 compiles several preparation methods to dope pristine graphene with nitrogen after its initial growth. Despite the necessity of post growth doping to create defects in the graphene lattice, a majority of the papers listed in Table 2 observed graphitic-N as the primary dopant bonding configuration.

There are three methods used in Table 2 to introduce nitrogen into a pristine graphene lattice: exposure to ammonia at elevated temperatures [17,34], bombardment with an ion gun [19,49,50], and exposure to nitrogen containing plasma [24,30,31,32,51,52,53,54,55]. Lv et al. prepared NG via the chemical reaction of graphene with ammonia directly after graphene growth [17]. The temperature in the reaction zone during the ammonia flow ranged from 750 to 950 °C, and it was found that the incorporation of nitrogen dopants in graphene changed depending on the reaction temperature and time. A low temperature (e.g., 750 °C) and short reaction time (e.g., 5 min), did not lead to any doping. In general, longer reaction times also lead to a less doped graphene as well. Nitrogen doping only occurred within a certain reaction temperature range (800–850 °C) and reaction times (10–30 min). The NG produced at 850 °C and 10 min contained 80% of a specific N dopant type—two graphitic-N dopants occupying next-nearest neighbor, adjacent A sublattice sites (N_2_^AA^), whereas the NG synthesized at 800 °C and 10 min, show more graphitic-N type dopants (single nitrogen substitution).

Nitrogen ion irradiation has also been used to dope graphene with nitrogen. In this method, nitrogen ions are accelerated by an electric field towards the target (graphene). Depending on the ion irradiation energy, different results including graphitic-N dopants, adatoms, and vacancy formation have been predicted by Åhlgren et al. [56] In their calculations a ~50 eV ion irradiation energy is optimal for introducing nitrogen atoms in the graphitic-N configuration, whereas lower and higher energy levels lead to adatom and vacancy formations, respectively. In this context, Cress et al. experimentally confirmed that 30–50 eV N^+^ ion energy is optimum to NG exhibiting the graphitic-N configuration [50]. It was also demonstrated that 46 eV N^+^ ion irradiation introduces dopants only in the top graphene layer of bilayer graphene. Bangert et al. carried out a more detailed investigation of the low N^+^ ion energy irradiation process [49]. At a N^+^ ion energy of 25 eV, 16% of the irradiated N^+^ ions are incorporated in the monolayer graphene lattice leading to a doping level of 1 at.% with 90% in the graphitic-N configuration [49].

Plasma treatment has been shown to be effective for doping carbon nanotubes with nitrogen [57,58,59]. Recent reports have also shown that this method can be used to dope graphene with nitrogen [24,30,31,32,51,52,53,54,55]. In general, when compared to thermal treatment, plasma treatment can introduce a higher concentration of nitrogen atoms. In terms of bonding configurations, Iyer et al. identified pyridinic-, pyrrolic-, nitrilic-, and graphitic-N configurations after the plasma treatment [32]. Rybin et al. reported that nitrogen plasma increased pyrrolic-N configurations due to the strong influence of ammonia radicals [53].

## 3. Characterization of Nitrogen Dopants in Doped Graphene

Not only can the dopant concentration affect graphene’s properties, but also the dopant bonding configuration. Among doped heterogenous graphene systems, nitrogen-doped graphene is the most investigated. As mentioned previously, substitutional nitrogen atoms in graphene primarily occupy either the graphitic-N, pyridinic-N, or the pyrrolic-N configurations (Figure 1c). Depending on the local configuration of the nitrogen atoms within the graphene lattice, the electronic, chemical, and catalytic properties of nitrogen-doped graphene will change. In this section we overview the techniques used to characterize dopant concentration and the different types of dopant configurations.

### 3.1. Dopant Concentration Characterization

The dopant concentration is one of the primary ways to characterize doped graphene systems. These systems are usually characterized by various spectroscopic techniques (Raman spectroscopy, XPS, X-ray absorption spectroscopy (XAS), energy dispersive X-ray spectroscopy (EDS), electron energy loss spectroscopy (EELS)), as well as several imaging techniques (scanning tunneling microscopy (STM), transmission electron microscopy (TEM)). Raman spectroscopy in particular is a technique used to characterize a wide variety of *sp*^2^-hybridized carbon allotropes such as graphene, carbon nanotubes, and fullerenes, in a non-destructive way under ambient conditions [60].

**Figure 3:**
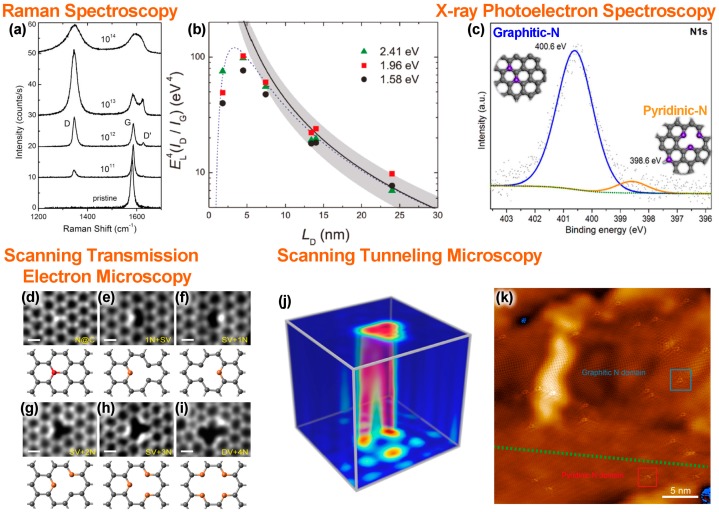
(**a**–**c**) Characterization of nitrogen dopant concentration and (**d**–**k**) dopant configuration. (**a**,**b**) Dopant concentration analyzed by Raman spectroscopy and (**c**) X-ray photoelectron spectroscopy (XPS). (**a**) In Raman spectroscopy the intensity ratio (*I*_D_/*I*_G_) of the disorder activated D-band (D) over the graphitic G-band (G) indicates the density of defects in NG. Reproduced with permission from [61]. Copyright Elsevier, 2010. (**b**) Evolution of *I*_D_/*I*_G_ against inter-defect distance *L*_D_ Reproduced with permission from [62]. Copyright American Chemical Society, 2011. (**c**) N 1s core-electron region X-ray photoelectron spectroscopy (XPS) spectra. Reproduced with permission from [17]. Copyright Springer Nature, 2012. (**d**–**k**) Dopant configuration has been characterized by scanning transmission electron microscopy (STEM) and scanning tunneling microscopy (STM). (**d**–**i**) Different nitrogen atom configurations identified using STEM. Nitrogen atoms possess higher Z-numbers so they appear relatively brighter than carbon atoms in STEM images. Reproduced with permission from [67]. Copyright American Chemical Society, 2015. (**j**) 3D STM map of tunneling current over a graphitic-N dopant (−100 mV, 1.8 × 1.8 × 0.15 nm^3^, *I*_set_ = 1 nA) and (**k**) STM image of segregated graphitic-N (blue) and pyridinic-N (red) domains. Reproduced with permission from [68] and [69]. Copyright American Chemical Society, 2015 and 2018, respectively.

In pristine graphene, there are two main peaks in the Raman spectra, a first-order ‘graphitic’ band (G-band, 1580 cm^−1^) (Figure 3a), and a two-phonon double resonance band (2D-band, 2670 cm^−1^). Dopants in graphene act as point defects similar to a vacancy or a topological defect. The impact of point defects on the Raman spectra of graphene has been systematically investigated. In this context, Lucchese et al. intentionally created point defects by irradiating mechanically exfoliated monolayer graphene with Ar^+^ ions (Figure 3a) [61]. Upon ion irradiation, a disorder activated band (D-band) emerges at 1350 cm^−1^. This D-band results from a one-defect and one-phonon double resonance process, and therefore indicates the presence of defects. By further increasing the defect density another defect activated D’-band (1620 cm^−1^) also emerges. Utilizing the intensity ratio of the ‘disorder’ and ‘graphitic’ bands (*I*_D_/*I*_G_), the crystallinity of the graphene-based system can be qualitatively determined. Since *I*_D_/*I*_G_ sensitively changes depending on the incident laser wavelength and defect density, Cançado et al. obtained a generalized equation for quantitative defect analysis [62]:

(1) $$n_{D}\left(cm^{-2}\right)=\frac{\left(1.8\pm 0.5\right)\times 10^{22}}{\lambda_{L}^{4}}\left(\frac{I_{D}}{I_{G}}\right)$$

where, $n_{D}$ and λ correspond to the defect density and the incident laser wavelength, respectively. *I_D_/I_G_* is graphed as a function of inter-defect distance in Figure 3b where inter-defect distance *L*_D_ is calculated from ion dose σ as $L_{D}=1/\sqrt{\sigma }$. When a certain dopant bonding configuration is assumed, the dopant concentration can be calculated from *L*_D_. Equation (1) is only valid for monolayer graphene. Jorio et al. also extended the work for multi-layered graphene [63]. In Raman spectroscopy all point defects act similarly, however, Eckmann et al. investigated the impact of different dopant types on the evolution of the defect activated bands. It was found that the *I*_D_/*I*_D’_ ratio sensitively changes depending on the type of point defect: an *I*_D_/*I*_D’_ ratio of ~7, ~9 and ~13 corresponds to *sp*^3^ bonding, substitutional dopants and vacancies, respectively [64,65]. Therefore, the *I*_D_/*I*_D’_ ratio can be used to identify the type of defects in graphene. In the case of CVD-grown NG, Zainab et al., reported that the *I*_D_/*I*_D’_ ratio was as low as ~3.5, a value which is similar to a boundary-type 1D defect [66]. In addition to Raman spectroscopy, other spectroscopy-based elemental analysis techniques—including XPS, EDS, and EELS—have been used to directly obtain the nitrogen/carbon atomic ratio. As discussed in the previous section, the dopant bonding configuration of nitrogen changes sensitively depending on synthesis conditions. Raman spectroscopy is not able to distinguish between different bonding configurations and other methods are needed in order to reveal the details of the incorporated dopants.

### 3.2. Dopant Bonding Configuration Characterization

The atomic configuration of dopants has been investigated by several spectroscopic and imaging techniques. Among them, XPS has been the de facto standard characterization method for identifying dopant configurations [15,70]. In an XPS spectrum, depending on the local configuration of dopants, different chemical shifts will be observed in the N 1s core-electron spectra (Figure 3c). Peaks located at around 398.3 eV, 400.1 eV, 400.2–401.8 eV, and 402.0–403.5 eV of the N 1s core-electron spectrum have been assigned to pyridinic-N, pyrrolic-N, graphitic-N, and oxidized-N, respectively. By combining XPS, core-level XAS and X-ray emission spectroscopy (XES), Schiros et al. reported that the major atomic configuration of nitrogen atoms in their ammonia-based nitrogen doped graphene is graphitic-N. By changing the ammonia partial pressure, or after transferring NG to a SiO_2_/Si substrate, pyridinic-N groups are found. Besides the pyridinic-N groups, a peak at around 398.5 eV has also been assigned to a nitrilic-N group, where a nitrogen dopant is bonded with one carbon and two hydrogen atoms [38]. Susi et al. also argued that the peak that has been assigned as pyrrolic-N could also be an N substitution in a Stone–Wales defect, or part of an amine, pyridone, nitroso, or cyano group [15].

One drawback of X-ray based techniques is its large spot size. In order to obtain atomic scale characterization of dopants, other methods with high spatial resolution can be used. By using an electron beam it is possible to focus the incident beam to a size comparable to atoms, thus allowing the atomic configuration to be directly observed. In this context, scanning transmission electron microscopy (STEM) has been used to directly observe the atomic configuration of nitrogen atoms. In particular, STEM-HAADF (high-angle annular dark field) enables Z-contrast imaging, which depends on the atomic number Z as Z^1.6–1.8^. The dopant atomic configurations of silicon [71], phosphorous [72], and germanium [14], have been revealed in this way. In STEM, electron energy loss spectra, collected by a local probe, is also useful when identifying heteroatoms and their dopant configurations [73]. Although low Z-number atoms are hard to identify under STEM, some sophisticated instruments can reveal the details of the atomic configuration of dopants [74]. For example, Lin et al. have investigated the atomic configuration of nitrogen atoms under STEM and several different types of dopant configurations were found (Figure 3d–i). Besides simple graphitic-N, SV+1N (single vacancy + pyridinic N), SV+2N (single vacancy + 2 pyridinic-N), SV+3N (single vacancy + 3 pyridinic-N), and DV+4N (divacancy + 4 pyridinic-N), were observed [67]. In their NG sample, each graphitic-N dopant is separated by at least 6.2 Å, while the active pyridinic-N sites were found to trap atoms. Using EELS, Lin et al. reported that transition metals (TM) such as Mg, Cr, Al, Mn, Ca, Fe and Ti, were bonding to nitrogen atoms in the pyridinic configuration within the graphene lattice [67]. When the nitrogen atom is incorporated in the pyridinic-N configuration, it lowers the N 1s level thus increasing the chemical reactivity. This concept has been used to realize atomically dispersed single-atom TM catalysts (Fe [75,76,77], Ni [78], Co [79,80], Cu [81,82], Ru [83]), within NG.

Besides STEM, STM can also reveal the atomic configuration of nitrogen dopants by investigating the local electronic properties (Figure 3j) [36]. In this technique the tunneling current between a metal tip and a sample is scanned over an area. When the nitrogen atom is embedded into the graphene lattice, the local density of states (LDOS) in the vicinity of the dopant will be modified. In constant current mode, this change in the LDOS changes the gap between the tip and doped graphene, thus the electronic fingerprint of the dopant will appear in a height mapping. For this technique the current, bias, and height between the tip and sample change the image, therefore theoretical calculations are important in order to identify the dopant bonding configurations [68]. When compared with STEM, the size of the topographic features resulting from the dopants observed in STM can be as large as 1.0 nm [18], which is one order of magnitude larger than the size of the atomic fingerprint (1–2 Å) of the dopants in STEM [74]. This allows STM to be used to investigate the spatial correlation between nitrogen dopants in low magnification images. Using STM to characterize NG, the graphitic N_2_^AA^ dopant configuration [17,19], sublattice segregation [16], single grain spatial segregation [21], and pyridinic-N/graphitic-N segregation (Figure 3k) [69] have been reported and identified.

## 4. Dopant Control in Nitrogen Doped Graphene

The above synthesis and characterization techniques describe the methods used to prepare and characterize nitrogen dopants in NG. Using the above characterization techniques, it has been shown that several types of dopant control have been achieved. Control of the dopant bonding configuration has been observed whereby a specific choice of synthesis parameters can promote growth of one type of dopant bonding configuration to be dominant relative to the others. Sublattice segregation of dopants has also been studied in graphitic-N samples, where dopants prefer to occupy a single triangular sublattice of graphene instead of being randomly distributed between the two. Another type of spatial segregation can be seen in NG where specific regions of a single graphene grain are observed to have a higher and lower density of dopants. In this section, we review these types of dopant control and their properties.

### 4.1. Dopant Bonding Configuration

**Figure 4:**
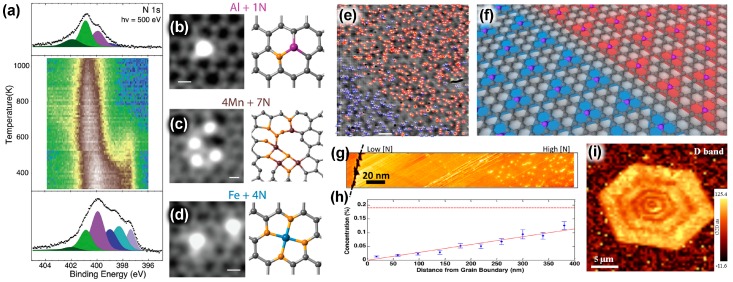
Nitrogen dopant control in NG. (**a**) Thermal evolution of the XPS N1s peak taken on Graphene/Ir(111). A shift to graphitic type dopants is seen as temperature rises. Reproduced with permission from [30]. Copyright Elsevier, 2016. (**b**–**d**) Scanning tunneling electron microscopy annular dark-field images of (**b**) Al, (**c**) Mn, and (**d**) Fe bonding to pyridinic-N sites in the graphene lattice. Scale bars are 2Å. Reproduced with permission from [67]. Copyright American Chemical Society, 2015. (**e**) Large-area STM image of NG on Cu(111). Nitrogen on different sublattices are marked by red and blue triangles. Scale bar is 10 nm. Reproduced with permission from [16]. Copyright American Chemical Society, 2014. (**f**) A model of nitrogen dopants occupying separate sublattices domains. (**g**) STM image of NG near a grain boundary indicated by the black line. (**h**) N-concentration as a function of the distance from the grain boundary in (**g**). The red dashed line is a linear fit to the data. Reproduced with permission from [21]. Copyright American Chemical Society, 2015. (**i**) Raman mapping of the D band intensity of a NG sample with a concentric hexagonal ring pattern. Reproduced with permission from [20]. Copyright Elsevier, 2018. The rings exhibit alternating low and high D-band intensity which indicate less and more N dopants respectively.

As mentioned previously, there are three main nitrogen bonding configurations reported in the literature when characterizing NG: graphitic-N, pyridinic-N, and pyrrolic-N. Studies have shown that thermal annealing can alter the dopant bonding configuration of nitrogen after the initial preparation of NG [30,84,85]. Orlando et al. synthesized NG by nitrogen plasma irradiation which resulted in pyridinic- and pyrrolic-N rich NG before annealing [30]. Using XPS, the authors observed the evolution of the N 1s spectra while ramping the annealing temperature from 300 K to 1040 K (Figure 4a). By analyzing the N 1s spectra, they observed that as temperature increased, the relative fraction of pyridinic-N and pyrrolic-N decreased while the relative fraction of graphitic-N increased due to its increased stability and the mobility of vacancies and dopants.

Different bonding configurations of nitrogen in NG will affect the local charge environment of neighboring atoms differently. They will also affect the global behavior of NG. Calculations indicate that nitrilic- and pyrrolic-N both p-type dope graphene, while graphitic-N leads to n-type doping [38,86,87]. Pyridinic-N can both n- and p-type dope graphene depending on whether or not the nitrogen dopant is bonded to hydrogen respectively [38]. This would not be obvious if one just considers the atomic Z-number, and it is important to keep in mind when using NG for device applications. For n-doping of graphene, the graphitic-N dopant is the most achievable type of dopant to use.

The local charge environment associated with the different dopant configurations in NG interacts with foreign atoms differently and leads to different catalytic activities. Several studies have looked into the catalytic activities towards the oxygen reduction reaction (ORR) of the main bonding configurations. Although there are conflicting accounts of a 2e reduction mechanism [25], NG has primarily shown a 4e pathway for the ORR [24,26]. Out of pyridinic-, pyrrolic-, nitrilic-, and graphitic-N, graphitic-N is shown to be superior when considering a single graphene layer for ORR [24,25,26]. In addition, calculations have shown that pyridinic-N is the best for the H_2_O_2_ reduction reaction [27]. As no single bonding configuration is best for all catalytic reactions, attention must be given to the bonding environment when studying NG applications in catalysis.

As previously mentioned, Lin et al. showed that specific bonding sites can preferentially adsorb TMs as a form of secondary doping of NG (Figure 4b–d) [67]. By selectively growing NG with pyridinic-N, these authors showed that TMs preferentially adsorb to these sites. This secondary doping can allow further tuning of graphene and enhance its electronic and catalytic properties.

### 4.2. Dopant Sublattice Segregation

Beyond controlling the nitrogen bonding configuration, when atomic resolution imaging is obtained in some NG samples with graphitic-N type dopants, nitrogen dopant sublattice segregation can also be observed (Figure 4e,f) [16,17,18,19]. In these samples, nitrogen atoms are observed to prefer only one of the graphene triangular sublattices, as opposed to being randomly distributed between the two sublattices. In Table 1 and Table 2 references which show sublattice segregation are marked with a star (*). There are two main proposed models for the mechanism behind this sublattice segregation. The first is an edge-growth model which proposes that during the growth of NG, specific edge sites of the graphene lattice are energetically favorable for nitrogen atoms in such a way that causes sublattice segregation [16,88]. The second model predicts that the sublattice asymmetry arises from inter-dopant interactions causing sublattice segregation to be energetically favorable over a random distribution [17,89,90].

A couple of issues can be brought up regarding the edge growth model. First is that if nitrogen is introduced to a specific sublattice via the edge during growth, then as different grains grow larger and join there should be well defined domains that coincide with the individual graphene grains [91]. This has not been seen experimentally. Second, by looking at Table 2 we observe examples of cases where the graphene is doped after the synthesis of pristine graphene, and sublattice segregation is also observed. This seems to directly invalidate the edge growth model as the only mechanism driving sublattice segregation although it could still play a part along with another model.

There are several predictions regarding new properties resulting from sublattice asymmetry in graphene. Firstly, spin-polarized transport is predicted to be achievable in this type of doped graphene [28,29]. In such a case, sublattice asymmetry is predicted to cause asymmetry in the band structure for electrons of different spins, enabling gating to shift the Fermi level to a point where it is in a large band gap for one spin but allowing transport via the other spin. Such a material would be useful for spintronic applications. Sublattice asymmetry is also predicted to maximize the band gap opening for a given concentration of nitrogen dopants which is a necessary requirement for many device applications [22,23]. Lastly, sublattice asymmetry is predicted to overcome the Klein tunneling effect [92], a phenomenon resulting from the Dirac dispersion of graphene’s electrons which causes electrons normally incident on a potential barrier to tunnel through with 100% efficiency [93]. Overcoming this effect would allow for confinement of electrons in graphene. These predictions have yet to be verified experimentally and provide an opportunity for future study.

### 4.3. Dopant Spatial Segregation

Spatial segregation of nitrogen dopants is another area that is beginning to be investigated. In this regard, Luo et al. revealed by Raman mapping and time-of-flight secondary ion mass spectrometry (TOF-SIMS) that nitrogen in NG is not homogeneously distributed and instead forms defect domains [25]. Later, using micro-Raman mapping, Zhao et al. were able to observe robust nitrogen dopant spatial segregation (Figure 4g,h) [21]. These authors observed that nitrogen atoms avoided graphene grain boundaries and edges, and that this phenomenon was independent of growth parameters including the choice of precursor, temperature, substrate, and pressure. This is in contrast with 3D materials, where impurities and dopants are known to migrate towards grain boundaries and surfaces during high-temperature annealing. Single crystal NG has also been shown to exhibit another unique type of dopant segregation (Figure 4i) [20]. By Raman mapping, Lin et al. observed concentric hexagonal rings of alternating N depleted regions and N rich regions in NG single crystals. The proposed mechanism is based on preferential nitrogen bonding to specific edge types of graphene. Since nitrogen bonds to zigzag edges more favorably than Klein edges, as the growth front of graphene changes from a zigzag edge to a Klein edge, regions of higher and lower nitrogen concentration, respectively, are obtained [20]. An explanation for the transition from one growth edge to the other is still not fully understood. These insights into the nitrogen dopant spatial heterogeneity provide new understanding of the nitrogen doping mechanism and emerging opportunities for tailoring the NG’s properties.

## 5. Conclusion and Perspective

**Figure 5:**
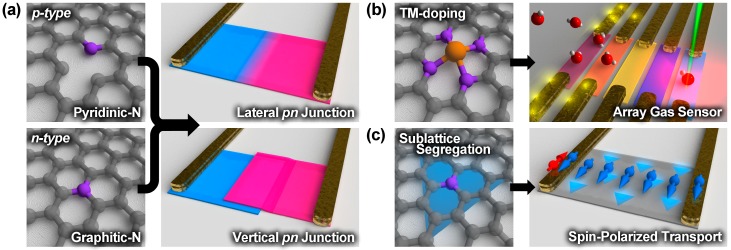
Possible device structures for NGs with different nitrogen dopant configurations. (**a**) Lateral and vertical p-n junctions for photodetection using pyridinic-N and graphitic-N as p- and n-type semiconductors, respectively. (**b**) Array gas sensor made by NG doped with several different transition metals (TMs). Differently colored sheets correspond to NG with different TMs. Different TMs act as the active sites and provide different sensitivity and selectivity to the sensor. By monitoring electric conductivity, optical signature, and the signal pattern, adsorbed gas type and concentration will be determined. (**c**) NG with sublattice segregated nitrogen dopants used as part of a spin polarized transport device.

Advances in growth control of nitrogen-doped graphene provide new opportunities and new understanding towards dopant-carbon interactions in graphene. This atomic scale control can allow NG to be optimized when constructing electronic/sensing devices. By tuning the nitrogen environment in NG, it is possible to tune its catalytic and sensing applications, as well as control p- and n-type electronic behavior. By combining p-type and n-type doped NG, 2D lateral and vertical p-n junctions could be created as possible photodetectors (Figure 5a) [94]. Pyridinic-N dopants facilitate the incorporation of various TM atoms and depending on the incorporated TM the interaction between the TM-modified NG and other molecules (e.g., gas molecules) changes [95,96]. By fabricating an array of NG domains doped with different TMs a graphene-based universal gas sensor can be realized in which the TM-modified electric/optical signal of NG can help to identify the type of gas (Figure 5b). Sublattice-segregated NG is predicted to have fascinating electrical properties that could be useful for spintronic applications (Figure 5c), and any device requiring a band gap. Spatial heterogeneity of dopants is beginning to be studied, and it is bringing an understanding of the growth mechanism of NG and other doped graphene systems. It is therefore clear that by understanding and controlling dopants in NG, emergent graphene applications will arise.

## Figures and Tables

**Table 1:** In situ NG preparation method and associated bonding configuration.

		CVD Conditions				Nitrogen Content (at.%)	Ref.
		Precursors	Pressure (Pa)	Substrate	Temperature (°C)		
**Major Nitrogen Dopant Configuration**	**Graphitic-N**	Methane + Ammonia	253	Polycrystalline Cu	1000	~0.3	[36,38]
		Methane + Ammonia	Atm	Polycrystalline Cu	800	8.9	[35]
		Methane + Ammonia	Unknown	Polycrystalline Cu	1000	N/A	[18] *
		Pyridine	2.7 × 10^−8^	Polycrystalline Cu	950	0.18	[16] *
		4,4,8,8,12,12-hexamethyl-8,12-dihydro4H-benzo[9,1]quinolizino[3,4,5,6,7-defg]acridine	<10^−7^	Pt(111)	400	0.4	[43]
		Hexamethylenetetramine	Atm	Polycrystalline Cu	1050	~0.6	[20]
		Pentachloropyridine	2000	Polycrystalline Cu	200–300	7.3–8.5	[44]
	**Pyridinic-N**	Ethylene + Ammonia	613	Polycrystalline Cu	900	0-16	[25]
		Quinoline	<10^−7^	Pt(111)	500	0.4	[42]
		Methane + Ammonia	Atm	Polycrystalline Cu	1000	~1	[37]
		Camphor + Melamine	Atm	Polycrystalline Cu	1015	2	[39]
		Melamine	Atm	Polycrystalline Cu	1000	8.9	[26]
	**Pyrrolic-N**	Pentachloropyridine	2000	Polycrystalline Cu	400–600	1.7–8.2	[44]
		Melamine	Atm	Polycrystalline Cu	1000	2.7	[26]
		Methane	low-pressure	Cu pretreated with NH_3_ plasma	1000	3	[45]
		Imidazole, PMMA	Atm	Polycrystalline Cu	1000	3.1	[46]
		Dimethylformamide	1	Polycrystalline Cu	950	3.4	[47]
		Methane + Ammonia	Atm	Polycrystalline Cu	880	4.56	[37]
		Nitrogen Gas + PDMS	3100	Polycrystalline Cu	700	~5.5	[48]

* indicate NG samples where sublattice asymmetry is observed.

**Table 2:** Post-growth treatment NG preparation method and associated dopant bonding configuration.

		Substrate	Post Growth Treatment	Annealing (°C)	Nitrogen Content (at.%)	Ref.
**Major Nitrogen Dopant Configuration**	**Graphitic-N**	Graphene/Cu	Ammonia exposure at 850 °C	N/A	0.25	[17] *
		Graphene/SiC(0001)	Nitrogen ion implantation	1027	0.13	[19] *
		Graphene/SiO_2_	10W plasma in N_2_ gas for 15 min	N/A	1.7	[33]
		Graphene/Cu	7eV nitrogen beam	N/A	2–3	[24]
		Graphene/O/Ir(111)	Nitrogen plasma	767	4	[30]
		Free-standing graphene	Nitrogen ion implantation	N/A	16	[49]
		Graphene/Cu	Nitrogen ion implantation	N/A	Unknown	[50]
		Free-standing graphene	Nitrogen plasma	N/A	Unknown	[32]
	**Pyridinic-N**	Graphene/Cu	12eV nitrogen beam	N/A	2–3	[24]
		Graphene/SiC (0001)	Neutralized nitrogen plasma	850	13.4	[31]
		Graphene/Ni	Ammonia plasma	800	Unknown	[54]
		Graphene/Ni foam	Ammonia exposure at 1000 °C	N/A	Unknown	[34]
	**Pyrrolic-N**	Graphene/SiO_2_	7W plasma in N_2_ gas for 20 min	N/A	1.8	[33]
		Graphene/SiO_2_	Plasma exposure in ammonia at room temperature	N/A	3	[53]

* indicate NG samples where sublattice asymmetry is observed.
